# Crystallization Modulation and Holistic Passivation Enables Efficient Two-Terminal Perovskite/CuIn(Ga)Se_2_ Tandem Solar Cells

**DOI:** 10.1007/s40820-024-01514-1

**Published:** 2024-09-22

**Authors:** Cong Geng, Kuanxiang Zhang, Changhua Wang, Chung Hsien Wu, Jiwen Jiang, Fei Long, Liyuan Han, Qifeng Han, Yi-Bing Cheng, Yong Peng

**Affiliations:** 1 State Key Laboratory of Advanced Technology for Materials Synthesis and Processing, Wuhan University of Technology, Wuhan, 430070 People’s Republic of China; 2 Triumph Photovoltaic Materials Co., Ltd., No. 1001 Yannan Avenue, High-Tech Development District, Bengbu, Anhui 233000 People’s Republic of China; 3 College of Materials Science and Engineering, Guilin University of Technology, Guilin, 541004 People’s Republic of China; 4 State Key Laboratory of Metal Matrix Composites, Shanghai Jiao Tong University, Shanghai, 200240 People’s Republic of China

**Keywords:** PVK/CIGS TSCs, Irregular rough surfaces, Modulating the growth, PVK/C60 interface recombination

## Abstract

Two-terminal (2-T) perovskite (PVK)/CuIn(Ga)Se_2_ (CIGS) tandem solar cells (TSCs) have been considered as an ideal tandem cell because of their best bandgap matching regarding to Shockley–Queisser (S–Q) limits. However, the nature of the irregular rough morphology of commercial CIGS prevents people from improving tandem device performances. In this paper, D-homoserine lactone hydrochloride is proven to improve coverage of PVK materials on irregular rough CIGS surfaces and also passivate bulk defects by modulating the growth of PVK crystals. In addition, the minority carriers near the PVK/C60 interface and the incompletely passivated trap states caused interface recombination. A surface reconstruction with 2-thiopheneethylammonium iodide and *N*,*N*-dimethylformamide assisted passivates the defect sites located at the surface and grain boundaries. Meanwhile, LiF is used to create this field effect, repelling hole carriers away from the PVK and C60 interface and thus reducing recombination. As a result, a 2-T PVK/CIGS tandem yielded a power conversion efficiency of 24.6% (0.16 cm^2^), one of the highest results for 2-T PVK/CIGS TSCs to our knowledge. This validation underscores the potential of our methodology in achieving superior performance in PVK/CIGS tandem solar cells.

**Supplementary Information:**

The online version contains supplementary material available at 10.1007/s40820-024-01514-1.

## Highlights

- Integrating perovskite solar cells onto irregular rough CuIn(Ga)Se_2_ (CIGS) surfaces remains a challenge; new strategy was explored to develop monolithic perovskite/CIGS tandem solar cell by manipulating the crystallization of perovskite.
- Surface reconstruction and field-effect passivation are developed synergistically to issue complex interface relationship between perovskite and C60.
- The champion  power conversion efficiency （PCE） of 24.6% realized, providing significant commercial opportunities for all thin-film-based perovskite/CIGS tandem cells.

**Supplementary Information:**

The online version contains supplementary material available at 10.1007/s40820-024-01514-1.

## Graphical abstract

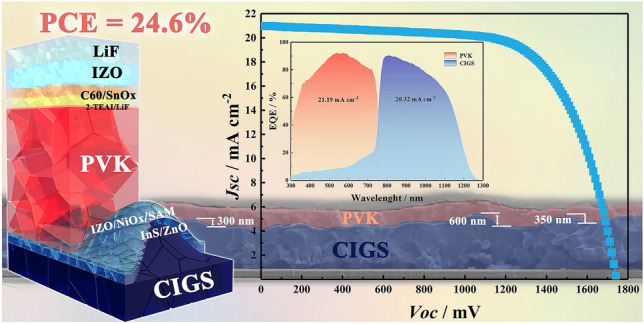

## Introduction

Two-terminal (2-T) perovskite (PVK)/CuIn(Ga)Se_2_ (CIGS) tandem solar cells (TSCs) have been considered as one of the most promising tandem technologies because of their bandgap matching capacity, regarding to Shockley–Queisser (S-Q) limit [1–3]. However, this tandem technology has been suffering from low performances in past years [4–8].

An obstacle of fabricating high-performance PVK/CIGS TSCs is the nature of the irregular rough surface morphology of commercial CIGS, which is typical lateral feature sizes in the order of 500 nm to 1 μm randomly distributing on CIGS surface [2, 7]. Consequently, incomplete surface coverage and shunt paths can easily take place while fabricating tandem devices [2, 9]. A chemical mechanical polishing interconnection layer strategy was used by Han et al. to smoothen surfaces, while Jošt et al. chose a highly technological co-evaporation method to prepare a uniform active CIGS surface [10, 11]. However, no whether polishing the interconnection layer or modifying CIGS preparing technology to obtain a smooth surface is costly and incompatible with industry. It is necessary to manipulate the deposition of top-cells. Normally, the growth orientation of the PVK crystals on the substrate is not under control, particularly in the case of more complex rough surfaces, resulting in disordered growth of grain. Strategies can be explored to manipulate the crystallization of PVK, to achieve the preferred orientation of grain growth to fully cover irregular (nano) rough surfaces [12–16]. In addition, incomplete passivation of trap states in PVK films and minority carriers near the interface is prone to trigger interface recombination, in turn reducing the efficiency of the device [17–22]. Growing the non-uniform PVK absorbers on the rough surface of CIGS resulted in more defects, which require further enhanced quality and mitigated recombination losses [23–26].

In this work, D-homoserine lactone hydrochloride (D-HLH) was involved to modulate PVK crystal growth, passivate bulk defects, and also improve the contacts of PVK films on CIGS surfaces as a consequence. In this instance, D-HLH has the capacity to form hydrogen bonds [–O–C(=O)···H–N and –NH···I–] with FAI, as well as robust Pb–O bonds with PbI_2_, which can control nucleation and crystal growth rates, and further improving crystal quality to cover irregular rough surfaces [27]. Furthermore, a surface reconstruction with 2-thiopheneethylammonium iodide (2-TEAI) and N, N-dimethylformamide (DMF) assisted passivates the defect sites located at the PVK surface and grain boundaries [18]. Simultaneously, LiF is used to create this field-effect, repelling hole carriers away from the PVK and C60 interface and thus reducing recombination [28]. Through this synergistic strategy, a 2-T PVK/CIGS tandem achieved 24.6% (0.16 cm^2^) of power conversion efficiency (PCE), which is one of the highest results for 2-T PVK/CIGS solar cells in our knowledge. This validation demonstrates the potential of our approach to achieve high PCE-values in PVK/CIGS tandem solar cells.

## Experimental Section

### Materials

Lead iodide (PbI_2_), lead bromide (PbBr_2_), cesium iodide (CsI), formamidine hydroiodide (FAI), methylammonium bromide (MABr), 2-thiopheneethylammonium iodide (2-TEAI), C60 (99.99%) and bathocuproine (BCP) were purchased from Xi’an Yuri Solar Co., Ltd. Lithium fluoride (LiF) was purchased from Sigma-Aldrich. [2-(9H-carbazol-9-yl)ethyl]phosphonic acid (2PACz) was purchased from TCI. D‑Homoserine lactone hydrochloride (D‑HLH) and Anisole were purchased from Macklin. The IZO and NiO target were purchased from ZhongNuo Advanced Material (Beijing) Technology Co., Ltd. All the chemicals were used as received without further purification.

### CIGS Solar Cells Fabrication

Triumph Photovoltaic Materials Co., Ltd., for providing products of commercial CIGS solar cells. CIGS is deposited via rapid-thermal processing; the substrate for the CIGS absorber(∼1.5 μm) is a soda-lime glass on top of which a molybdenum (Mo) back contact is deposited via a direct-current (DC) magnetron sputtering. After the absorber deposition, the CIGS solar cells are completed with a 40-60 nm InS (Indium Sulfide) and a 60 nm i-ZnO (intrinsic zinc oxide).

### Single-Junction Perovskite Solar Cells Fabrication

The ITO glass substrates underwent a sequential cleaning process, which involved sonication in detergent, deionized water, and ethanol for durations of 15 min each. Substrates were subsequently dried using N_2_ flow and exposed to ultraviolet-ozone treatment for a duration of 15 min. A self-assembled monolayers (SAM) of 2PACz (1 mg mL^−1^ in absolute ethanol) was spin-coated at 3000 rpm for 30 s on ITO and subsequently annealed at 100 °C for 10 min in N_2_ glovebox. Next, 1.4 M Cs_0.05_(FA_0.83_MA_0.17_)_0.95_Pb(I_0.83_Br_0.17_)_3_ PVK precursor solution was prepared by dissolving a mixture of FAI, MABr, CsI, PbI_2_, and PbBr_2_ in a mixed DMF:DMSO (4:1 v/v). D-HLH was added to the precursor solution at optimized concentrations (0.5 mg mL^−1^). The PVK films were spin-coated at 2000 rpm for 45 s, then followed with 7000 rpm for 10 s. Anisole of 300 μL was dropped in the center of the substrates 12 s before the end of the spin-coating process. Subsequently, the substrates were immediately transferred onto a hotplate of 100 °C and were annealed for 15 min. For the devices with surface passivation treatment, 2-TEAI (1 mg mL^−1^) was dissolved in a mixed solvent of IPA:DMF (150:1, v/v), spun onto the as-prepared PVK films at 5,000 rpm for 30 s, and then annealed at 100 °C for 5 min. After cooling to room temperature, the annealed films were washed by 100 µL IPA with the same spin coating procedure mentioned above, and then the washed film was transferred to a 100 °C hot plate and dried for 3 min. Sequentially thermally deposited LiF (1 nm), C60 (25 nm), BCP (6 nm) and silver (100 nm) to complete the device.

### PVK/CIGS Tandem Solar Cell Fabrication

The CIGS cell fabrication process for 2 T TSCs is followed as described before up to the i-ZnO_x_ layer. After that, a 20 nm of indium-doped zinc oxide (IZO) is sputtered to serve as recombination layer. As hole transporting layer, nanocrystalline NiOx with a thickness of ∼15 nm was sputtered from NiO target (99.9%) in an Ar atmosphere using Angstrom Engineering EvoVac system. The 2PACz, PVK, LiF and C60 (15 nm) layers are deposited in the same way as with the single-junction perovskite solar cells (PSCs). Instead of BCP, a 20 nm of SnO_2_ was deposited by atomic layer deposition (ALD) in an Arradiance GEMStar reactor. A transparent front electrode (70 nm of IZO) is sputtered from a IZO ceramic target on top of the SnO_2_ through a shadow mask to overlap of the Mo back electrode at 43 W. Ag finger with a thickness of 300 nm was thermally evaporated using shadow mask. Finally, 100 nm LiF was thermally evaporated as an anti-reflection layer.

### Characterizations

The surface and cross-sectional morphology of the PVK films were acquired by scanning electron microscopy (SEM) (QUA TTROS, Thermal Fisher Scientific). ^1^H NMR and ^13^C NMR spectra were collected using a Bruker 400/600 MHz instrument Spectrometer. Fourier Transform Infrared Spectroscopy (FTIR) was performed by Thermo Scientific Nicolet iS20. X-ray photoelectron spectroscopy (XPS) measurement was carried out on a Thermo Scientific ESCALAB Xi + system with a monochromatized Al Kα (for XPS mode) under a pressure of 5.0 × 10^−7^ Pa. X-ray diffraction (XRD) data were obtained by using a Bruker D8 Advance diffractometer. The surface morphology of the PVK film was collected by atomic force microscope (AFM) (Park XE7). The PL spectra were obtained using a PL microscope (Flex One, Zolix, China). Time-resolved PL measurements were conducted using a Delta Flex Fluorescence Lifetime System (Horiba Scientific Com, Japan) under excitation at 470 nm light pulse. C-V curves were measured using an EC-lab (SP300) instrument. The *J-V* curves were measured under AM 1.5 G sunlight (100 mW cm^−2^), using a solar simulator (Oriel 94023A, 300 W) and Keithley 2400 source meter. The *J-V* measurements were conducted with a scan rate of 100 mV s^−1^ (voltage step of 10 mV and delay time of 100 ms) from open voltage to short circuit in forward (−0.1 to 1.2 V) and reverse (1.2 to −0.1 V) directions at room temperature in the air. A mask with an aperture area of 0.16 cm^2^ for tandem device was used. No preconditioning protocol, such as light soaking or forward voltage bias applied for a long time, was used before starting the measurement. The steady-state PCE was determined by the time series of power output at the maximum power point voltage extracted from the *J-V* curves. For the maximum power point stability testing, encapsulated devices were exposed to continuous illumination by a simulated AM 1.5G one-sun illumination in open ambient air. External quantum efficiency (EQE) spectra of devices were measured using a solar cell quantum-efficiency measurement system. Photoluminescence quantum yield (PLQY) was measured by Enli Tech, with a 405 nm laser light source.

## Results and Discussion

### Crystallization Modulation and Holistic Passivation

**Fig. 1:**
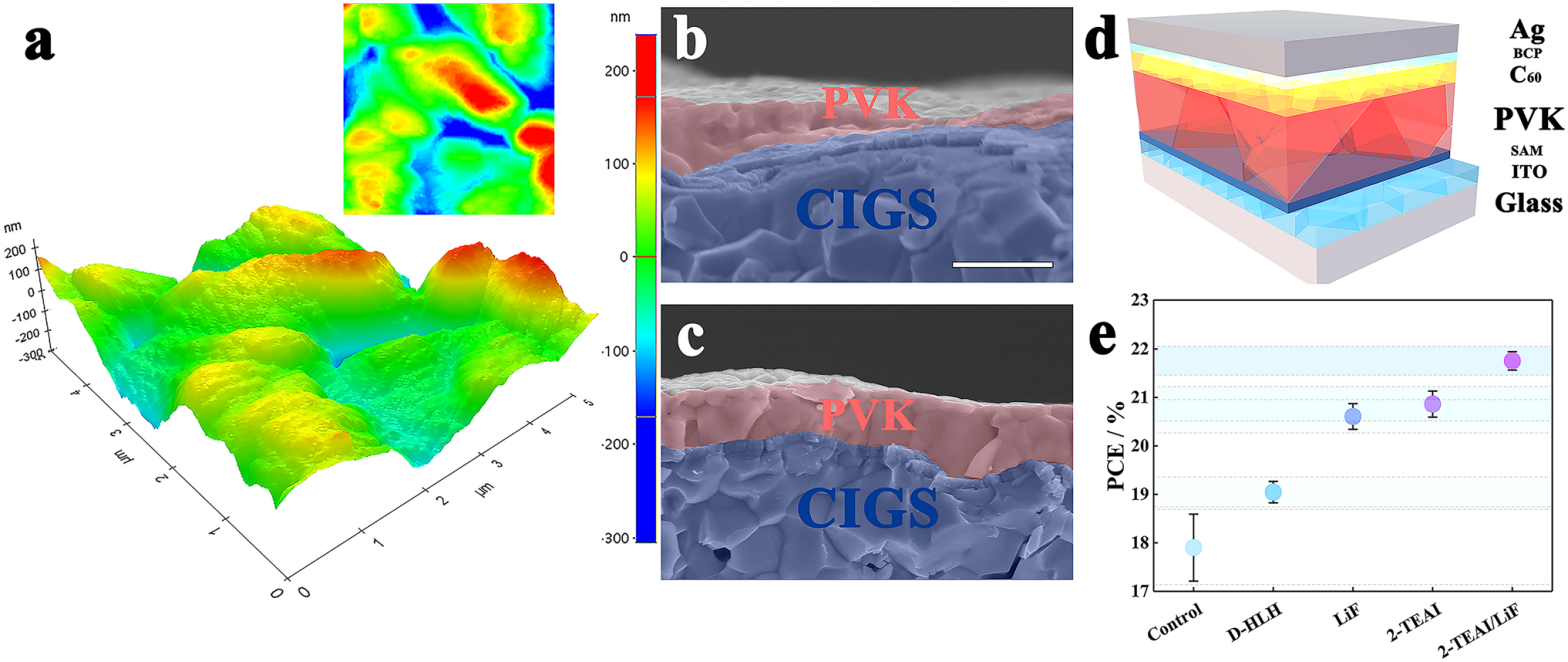
Characteristics of the CIGS and p-i-n structured PSCs. **a** 5 µm × 5 µm AFM topography of surface CIGS. Cross-sectional SEM images of PVK fabricated by **b** control and **c** additive engineering in PVK/CIGS tandem devices. (Scale bar: 1 μm) **d** Architecture of the single junction PSC device. **e** PCEs of control versus treated PSCs using different strategies

An irregular (nano) rough (variation of ≈550 nm) commercial CIGS cell surface remains a key challenge for integrating the PVK sub-cell onto the top, as shown in Fig. 1a. Several hurdles hinder the achievement of high-performance devices: uncovered surface of CIGS (Fig. 1b), shunt paths, and inefficient charge collection in films. A PVK composition of Cs_0.05_(FA_0.83_MA_0.17_)_0.95_Pb(I_0.83_Br_0.17_)_3_ (~ 1.63 eV) was applied with an additive engineering strategy to obtain reasonable surface coverage of CIGS. As anticipated, cross-sectional scanning electron microscope (SEM) reveals that the surface of CIGS is entirely coated with PVK film (Fig. 1c), and the irregular crystals transformed into vertical-growth monolithic grains with compact and dense morphology, resulting in a reduction in surface roughness (Fig. S1). Meanwhile, the introduction of D-HLH into PVK precursors can passivate bulk defects. On this basis, we combined surface/interface dual passivation to address the issue of carrier recombination at complex PVK/C60 interface. An effective surface reconstruction with 2-TEAI and DMF-assisted, which passivates the defects sites on located at the PVK surface and grain boundaries. And LiF as interlayer can provide effective field-effect passivation between PVK and C60 interface. We explored the impact of these efforts on the performance of single-junction PSCs. The device architecture consists of ITO/SAM/PVK/C60/BCP/Ag (Fig. 1d). The additive engineering enabled a device performance improvement compared with the control devices, from average PCEs of ~ 17.9% to ~ 19.1% (Figs. 1e and S2). In comparison with single surface or interface passivated devices, the highest average PCE (> 21.8%) is achieved with 2-TEAI/LiF combination (Figs. 1e and S2).

### Mechanism of Modulated PVK Films Crystal Growth

**Fig. 2:**
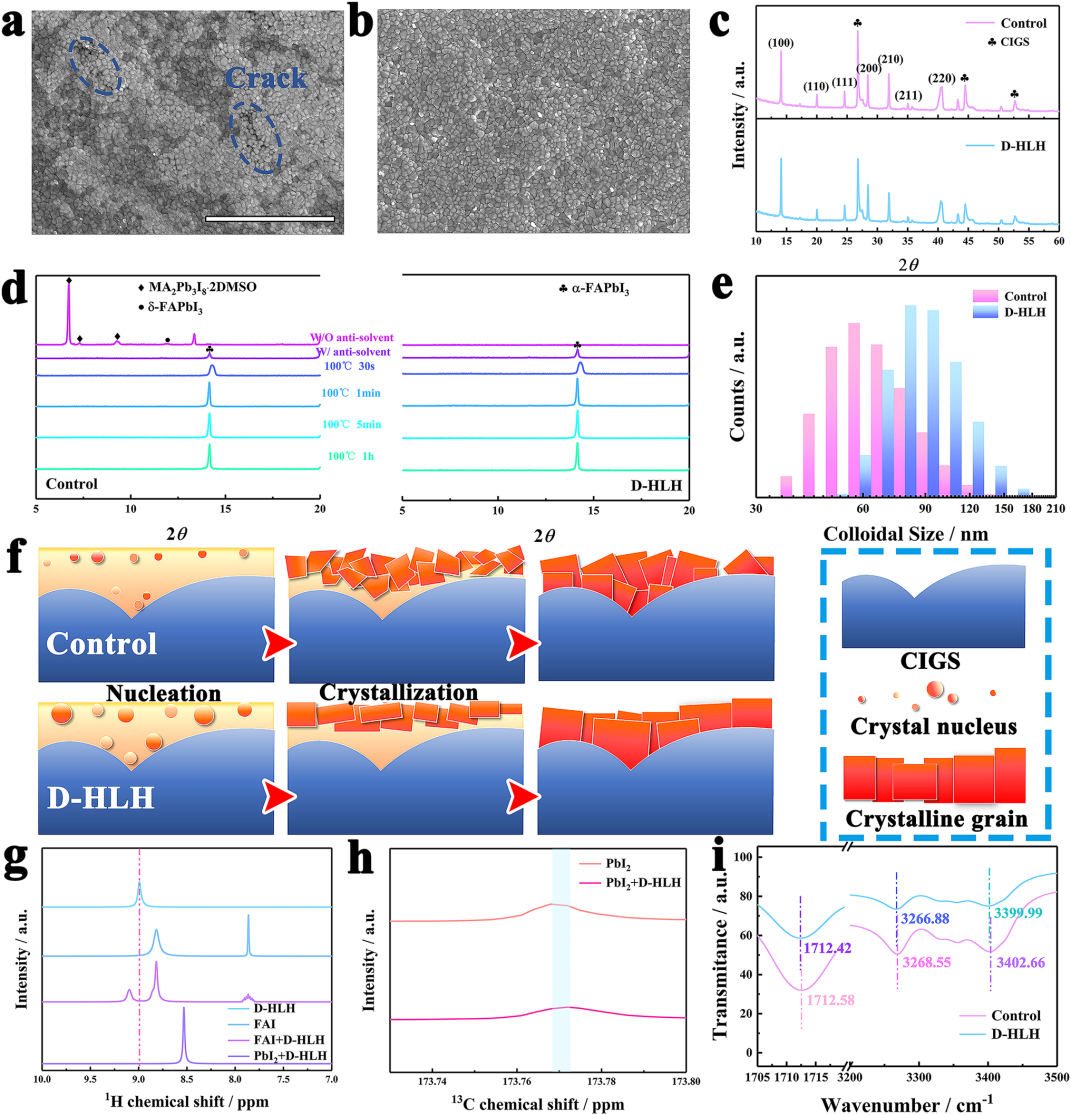
Mechanism of the modulated crystal growth by additive. **a** Top-view SEM images of PVK of control and **b** D-HLH-treated as-grown on CIGS (Scale bar: 5 μm). **c** XRD patterns of the control and D-HLH-tread PVK films as-grown on CIGS. **d** Tracking of X-ray diffraction of the PVK films during three processes: wet PVK film without (w/o) antisolvent during spin-coating, wet PVK film with (w) anti-solvent during spin coating, and PVK films annealed at 100 °C for various times. **e** DLS spectra of control and D-HLH incorporation into precursor solutions. **f** Schematic diagram of the crystal growth process of control and D-HLH-based precursor solutions on the surface of CIGS. **g** ^1^H NMR spectra of D-HLH, FAI, FAI/D-HLH, and D-HLH/PbI_2_. **h** ^13^C NMR spectra of PbI_2_ and PbI_2_/D-HLH. **i** FTIR spectra of the control and D-HLH-tread PVK films

In comparison with the non-continuous contact of the control, better coverage of the PVK film on the CIGS surface was observed in the top-view SEM images after the incorporation of D-HLH into the precursor, as anticipated (Fig. 2a, b). Furthermore, the enhancement of film quality and coverage on the CIGS substrate is also verified by the increased PVK peak (14.0°) intensity of (100) plane and the decrease in intensity of the CIGS peak in the X-ray diffraction spectroscopy (XRD) pattern (Fig. 2c). The peak intensity ratios of (100)/(110) and (100)/(111) were subsequently calculated and recorded as 3.22 and 2.75 (control), as well as 3.91 and 3.16 (D-HLH). The addition of D-HLH resulted in an increase in the peak intensity ratio, suggesting a preferential orientation along the (100) direction. It is hypothesized that D-HLH can modulate the crystal growth of PVK, which aids achieve good crystallinity and coverage on rough surfaces.

Subsequently, we examined the impact of additives on the nucleation and crystallization process of PVK, a process that typically occurs swiftly during spin-coating and initial annealing. To ascertain this transformation process, XRD analysis was conducted (Fig. 2d). In the wet PVK film, complex intermediate phases such as MA_2_Pb_3_I_8_⋅2DMSO (2*θ* = 6.73°, 7.38°, 9.33°) and δ-FAPbI_3_ (2*θ* = 11.9°) were formed in the control PVK due to the robust interaction between the PVK precursor and DMSO [13]. Upon the incorporation of D-HLH, the wet PVK films manifested amorphous characteristics without discernible crystallization, attributable to the hydrogen-bonding interaction between D-HLH and the PVK precursors. Subsequent to the application of the anti-solvent, a more pronounced peak intensity of α-FAPbI_3_ was observed compared to the control, suggesting that the film based on D-HLH demonstrates a higher nucleation rate relative to the control film. Consequently, dynamic light scattering (DLS) was employed to investigate the nucleation of the PVK precursor solution, as shown in Fig. 2e. The DLS peak center exhibited a shift from 65.55 to 91.01 nm in comparison with the control. The existence of larger colloids suggests that pre-nucleation has transpired in the PVK precursor solution introduced by D-HLH. This process lowers the energy barrier for nucleation during precipitation, thereby expediting the transition from an intermediate phase to preformed PVK nuclei. With annealing at 100 °C, the peak of α-phase rose and the width of the half-peak gradually narrowed. Moreover, as the annealing time progresses (0–30 s), the target film exhibits a relatively slower growth rate compared to the control film (Fig. S3). In conclusion, the D‑HLH treatment inhibited the formation of a mixed intermediate phase by converting the diverse intermediate adducts into pure α-phase PVK nuclei during both nucleation and crystal growth stages. Meanwhile, higher nucleation rates and slower crystal growth with preferential orientation result in better film coverage on irregular rough CIGS surfaces. As illustrated in Fig. 2f, microcrystals grow rapidly in an oversaturated solution and stack randomly. This leads to the formation of discontinuous PVK films on the irregularly rough CIGS surface due to the random orientations of the growing PVK. In contrast, when the D-HLH additive is incorporated, optimal nucleation of PVK is achieved, along with slower crystal growth and preferential orientation, thereby achieving higher quality and vertically oriented PVK films to fully cover the irregular rough surface of CIGS.

The liquid-state proton nuclear magnetic resonance (^1^H NMR) measurement was conducted to investigate the chemical interaction between additives and PVK precursors. This interaction dictates the formation of intermediate phases, thereby significantly influencing the crystallization process. In a pure deuterated DMSO solution, the ^1^H resonance signal at 8.82 ppm, which originated from protonated ammonium in neat FAI, was split into two peaks at 8.95 and 8.82 ppm in the D-HLH + FAI system. Concurrently, the single peak at 7.878 ppm was divided into multiple peaks, suggesting the occurrence of a hydrogen bonding interaction between D-HLH and FA^+^ ions (Fig. 2g). To substantiate this interaction, the pure D-HLH and the mixed solution of D-HLH + FAI were characterized using ^13^C NMR. As depicted in Fig. S4, the ^13^C NMR spectrum at 173.768 ppm of the pure D-HLH corresponds to the ester group. This group also experiences a corresponding shift in the mixed solution of D-HLH + FAI, thereby reinforcing the interaction between FAI and D-HLH. In addition, when PbI_2_ was incorporated into the D-HLH solution, the ^1^H peak of -NH_3_^+^ at 9.03 ppm was shifted to 8.53 ppm, implying that there also was an interaction between PbI_2_ and the D-HLH, a larger shift amplitude implies that D-HLH had a stronger interaction with PbI_2_ as compared to FAI (Fig. 2g). At the same time, it is found that the ^13^C NMR peak is shifted to 173.772 ppm after the introduction of PbI_2_, which originates from the interaction between polar ester [–O–C(=O)–] and uncoordinated Pb^2+^ (Fig. 2h). These results confirm that D-HLH can interact with the FAI and PbI_2_, which affect its reaction rate, eventually modulating the PVK crystallization. Additional evidence of the interaction was obtained through Fourier transform infrared spectroscopy (FTIR) and X-ray photoelectron spectroscopy (XPS). In the FTIR spectra (Fig. 2i), the peaks corresponding to N–H stretching and C=O stretching shifted to lower wavenumbers following the incorporation of D-HLH, indicating an interaction between the PVK and the D-HLH molecule. The chemical environment of various components within the PVK was also examined using XPS (Fig. S5). In the case of Pb^2+^ ions, the introduction of D-HLH led to a shift in the Pb 4*f* signal toward higher binding energies. This suggests that the ester group within D-HLH formed strong Pb–O bonds with the Pb^2+^, resulting in a reduction in electron density on the Pb atoms. The observed shift in the I 3*d* peaks is attributed to the hydrogen bond (–N–H···I^–^) formed between the polar amino group of D-HLH and the electronegative iodide. Furthermore, the shift in the N 1*s* signal as evidenced by XPS indicates that D-HLH has the capacity to form hydrogen bonds with the amino groups in FA^+^. This interaction plays a crucial role in mitigating the loss of organic cations and reducing the density of defect states [29].

### Effect Mechanism of the Surface/Interface Dual Passivation

**Fig. 3:**
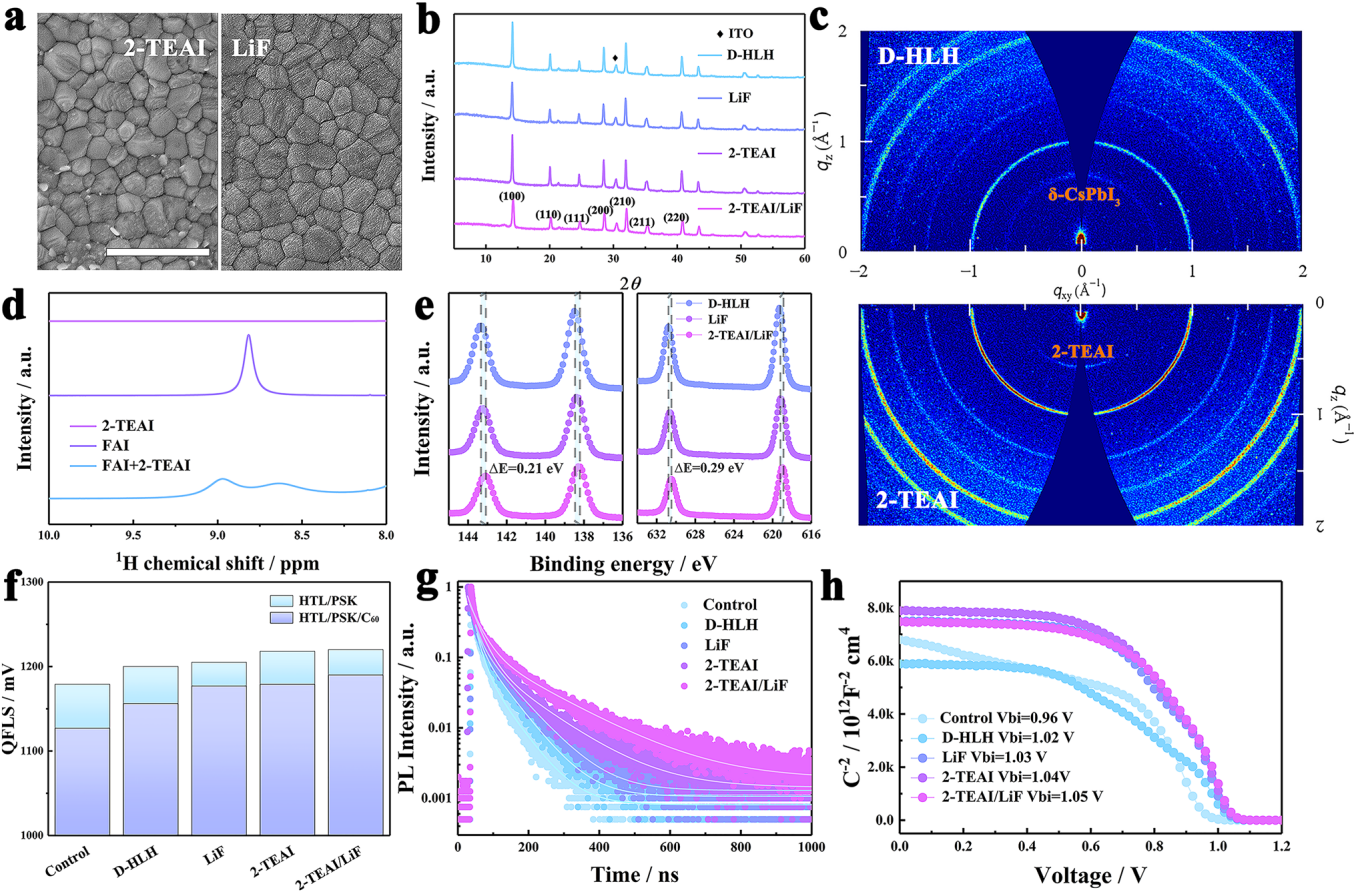
Mechanism of surface/interface dual passivation. **a** Top-view SEM images of PVK surface with 2-TEAI and LiF-treated (Scale bar: 1 μm). **b** XRD pattern of PVK films. **c** GIWAXS patterns of the D-HLH-based and 2-TEAI-treated PVK films. **d** ^1^H NMR spectra of 2-TEAI, FAI, and FAI with 2-TEAI. **e** High-resolution Pb 4*f* and I 3*d* XPS peaks of the PVK films. **f** Quasi-Fermi level splitting (QFLS) of films deposited on glass/ITO/SAM without and with C60. **g** Transient PL spectra for the glass/PVK samples. **h** Mott−Schottky plots for the corresponding devices

The alterations in surface morphology resulting from various surface-treatment processes were validated using top-view SEM (Fig. 3a). The morphology of the PVK films remained almost unchanged for the 2-TEAI-treated, while after LiF evaporation, LiF clusters are formed on the surface of the PVK film. Meanwhile, the XRD patterns of PVK are nearly identical, and no clear two-dimensional (2D) PVK phase is observed (Fig. 3b). Surface-sensitive grazing-incidence wide angle X-ray scattering (GIWAXS) further revealed a peak at low scattering vectors q ≈ 0.6 Å^−1^ (2*θ* = 8.7°) in the 2-TEAI-based film, which corresponds to the 2-TEAI powder or film, indicating no low-dimensional PVK formed (Fig. 3c) [30]. Meanwhile, DMF-assisted reconstruct the PVK surface, providing more active A-site available for 2-TEA cations substitution can significantly enhance the PVK crystal quality and eliminate non-perovskite yellow phase δ-CsPbI_3_ (q ≈ 0.71 Å^−1^) at the surface [31].

^1^H NMR spectra (Fig. 3d) also showed that the amino proton peak of FAI at 8.82 ppm was split into two broader peaks after mixing with 2-TEAI. These changes indicated stronger hydrogen bonding interactions between 2-TEAI and FA^+^ ions. Due to such a strong interaction, 2-TEAI is likely to attach at the A sites in an ABX_3_ PVK. We assessed surface passivation using XPS, as shown in Fig. 3e. XPS data of only LiF-coated PVK showed that the chemical environment of lead and iodine is not appreciably modified because fluoride ions did not form strong chemical bonds with lead. However, with dual surface/interface passivation, the Pb 4*f* peaks of the passivated PVK film shifted toward a lower binding energy of 0.21 eV compared with the D-HLH-based film, which could be due to ionic bond between 2-TEA^+^ and PbI_6_^4–^, hydrogen bond of 2-TEA^+^ with I^–^, and/or coordination between S and Pb^2+^.

To demonstrate the effect of surface or interface treatment, we quantified the nonradiative recombination loss at the PVK/C60 interface using quasi-Fermi level splitting (QFLS). We measured the photoluminescence quantum yield (PLQY) of PVK films on glass/ITO/SAM with and without C60 deposition (Fig. S6). The corresponding QFLS values are 1179, 1200, 1205, 1218, and 1220 mV for control, D-HLH, LiF, 2-TEAI, and 2-TEAI/LiF samples, respectively (Fig. 3f). The significant changes in QFLS indicate the effectiveness of D-HLH and 2-TEAI in passivating bulk and surface PVK defects. However, the introduction of the C60 layer caused significant interfacial non-radiative recombination in the control stack. The LiF-induced field-effect effectively suppressed non-radiative recombination. The sequential combination of 2-TEAI/LiF further eliminated it, as confirmed by the improvement of QFLS.

We conducted further evaluations on the carrier recombination kinetics of PVK films. The steady-state PL spectra indicated that non-radiative recombination was significantly inhibited (Fig. S7). The 2-TEAI/LiF surface/interface dual passivation demonstrated a higher PL emission intensity compared to single surface or interface passivation. And the fitted PL lifetime by transient PL is increased from 67 (control) to 79 (D-HLH), 81 (LiF), 106 (2-TEAI) and 152 ns (2-TEAI + LiF), respectively, after the corresponding passivation (Fig. 3g). Treatment with LiF showed little improvement in lifetime, reflecting its limited suppression of defect-induced surface recombination. Conversely, the PVK film subjected to 2-TEAI treatment exhibited a marked enhancement in the decay curve, indicative of an extended carrier lifetime attributable to the diminishment of surface defects. This trend indicates that the surface passivation of 2-TEAI and field-effect passivation of LiF can achieve the best surface/interface passivation. The variation in interfacial charge transfer rate subsequent to surface or interface passivation is manifested in the transient PL lifetime of the PVK/C60 stacks (Fig. S8). The results indicated that the PL lifetime decreases from 23.6 (control) to 19 (D-HLH), 18.4 (LiF), 17 (2-TEAI) and finally to 15.9 ns (2-TEAI/LiF).

The enhancement of the built-in potential (Vbi) further corroborated effective charge separation and mitigated non-radiative recombination, thereby yielding a high output voltage. Utilizing the Mott–Schottky relationship, the capacitance–voltage (*C-V*) curve was meticulously measured to scrutinize the Vbi of the PVK film. As shown in Fig. 3h, the Vbi value of different devices increased from 0.96 (control) to 1.02 (D-HLH), 1.03 (LiF), 1.04 (2-TEAI) and 1.05 V (2-TEAI + LiF).

### 2-T PVK/CIGS Tandem Solar Cells

**Fig. 4:**
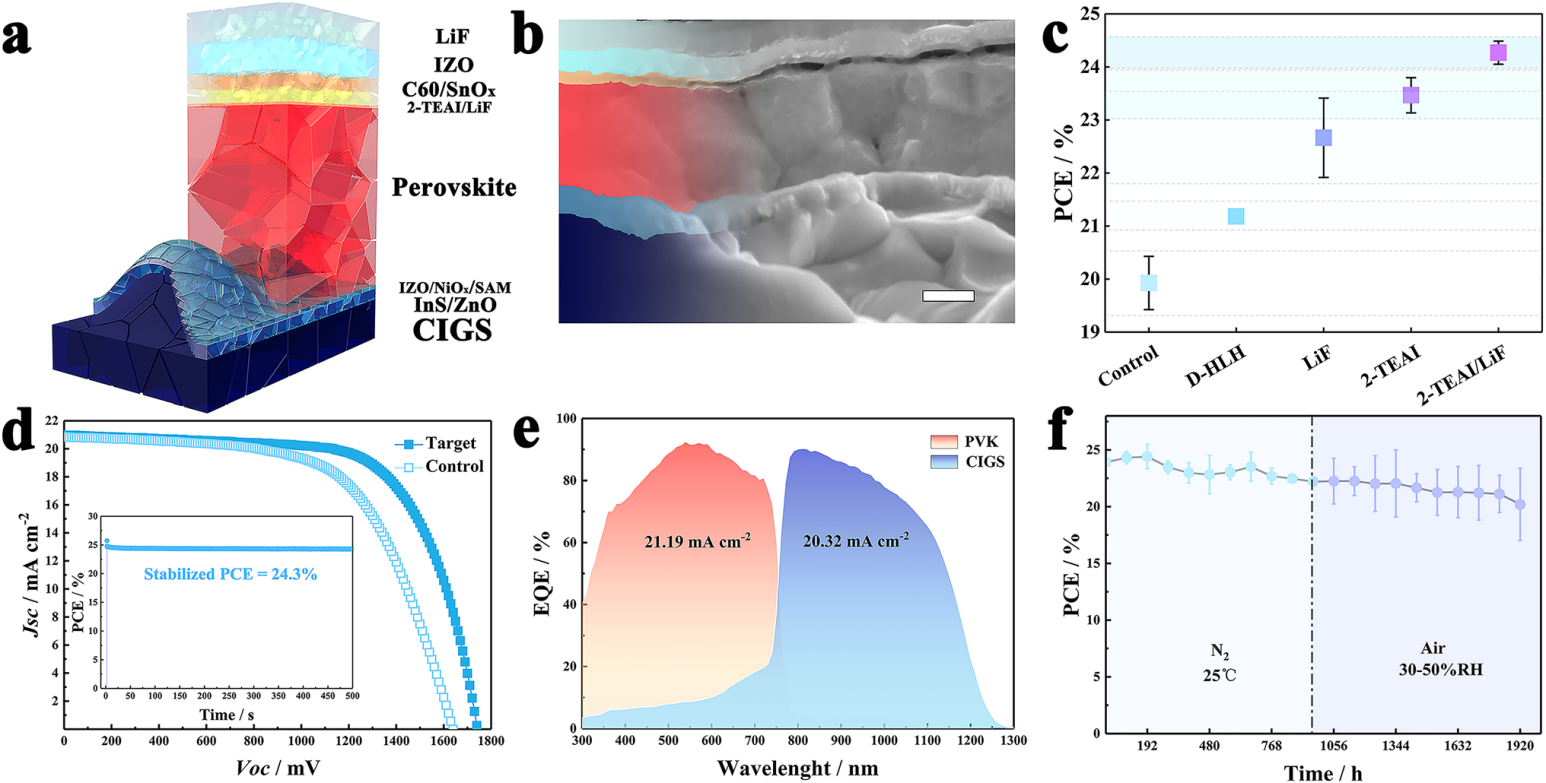
Performance of the PVK/CIGS tandem solar cell. **a** Schematic structure of the 2-T PVK/CIGS tandem device. **b** Cross-sectional SEM image of the tandem device (Scale bar: 200 nm). **c** PCEs of control versus treated tandem devices using different strategies. **d** *J-V* characteristics of the best-performing tandem device with control and target. The inset shows the stabilized PCE of the champion device under maximum power point tracking. **e** EQE curves of PVK and CIGS sub-cells within the tandem device. **f** Stability of unencapsulated tandem devices in nitrogen to air

Inspired by the enhanced performance of single-junction PSCs, we developed 2-T PVK/CIGS tandem solar cells with the aim of enhancing efficiency and pushing the boundaries of the future photovoltaic market. Figure 4a illustrates a schematic of the tandem configuration stack, and the structure of tandem device is Glass/Mo/CIGS/InS/i-ZnO/IZO/NiO_x_/SAM/1.63 eV PVK/LiF/C60/ALD SnO_2_/IZO/Ag. Here, a 20 nm indium zinc oxide (IZO) layer acted as the recombination junction, while the 15-nm-thick sputtered NiO_x_ layer between the IZO-SAM interface effectively reduced shunting, and the full layer stack and layout for our 2 T PVK/CIGS TSCs are depicted in Fig. S9. Cross-sectional SEM images of tandem solar cells are shown in Figs. 4b and S10. Figure 4c presents the current density–voltage (*J–V*) curves of the best-performing different treated PVK/CIGS tandems; the corresponding photo‑voltaic parameters are summarized in Figs. S11 and S12. The champion PVK/CIGS tandem device achieved a remarkable reverse-scan PCE of up to 24.6%, with a short-circuit current density (*J*_*SC*_) of 20.97 mA cm^−2^, open-circuit voltage (*V*_*OC*_) of 1.742 V, and fill factor (FF) of 67.3%, whereas the control device showed the highest PCE of 20.9%, with a *J*_*SC*_ of 20.84 mA cm^−2^, *V*_*OC*_ of 1.640 V, and FF of 61.2%, (Fig. 4d). And external quantum efficiency (EQE) spectra (Fig. 4e) exhibited a matching current density of 21.19 and 20.32 mA cm^−2^ in PVK and CIGS sub-cells, respectively. The steady power output of champion tandem devices (*Vmax* = 1.36 V) reached 24.3% after continuous illumination at 1 sun-simulated light for 500 s (Fig. 4d). Our tandem device is commensurate with the state-of the-art 2-T PVK/CIGS tandem devices in literature (Table S1). In the study of the stability of unencapsulated tandem devices, *J-V* is regularly measured (Figs. 4f and S13). After 960 h of nitrogen storage, the cells maintained 92.8% of their initial efficiency. Subsequently, after being exposed to an air environment with a humidity level of 30%–50% for the same period, its efficiency remained at 84.4% of the initial efficiency.

## Conclusions

Controlling the crystal growth of PVK to cover the rough surface of CIGS, while further improving crystal quality. Surface reconstruction passivates the defects sites located at the surface and grain boundaries. Meanwhile, creating a field-effect that repels hole carriers outside the interface between PVK and C60 reduces recombination. Realizing both bulk and surface/interface passivation by the combined use of D-HLH, 2-TEAI, and LiF has mitigated PVK crystallization and complex interface carrier recombination issues. Through this synergistic strategy, a 2-T PVK/CIGS tandem achieved 24.6% (0.16 cm^2^) of PCE. The amalgamation of various functional regulation techniques presents a promising avenue for investigating the performance and stability of PVK-based tandems.

## Supplementary Information

Below is the link to the electronic supplementary material.

Supplementary file 1
