# Supplementary material for: Crystallization Modulation and Holistic Passivation Enables Efficient Two-Terminal Perovskite/CuIn(Ga)Se2 Tandem Solar Cells
Source: Nanomicro Lett. 2024 Sep 22;17:8. doi: 10.1007/s40820-024-01514-1 (PMC11416436; doi:10.1007/s40820-024-01514-1)
Supplement: Supplementary file 1 [file 40820_2024_1514_MOESM1_ESM.docx]

Supporting Information for

**Crystallization Modulation and Holistic Passivation Enables Efficient Two-Terminal Perovskite/CuIn(Ga)Se_2_ Tandem Solar Cells**

Cong Geng^1^, Kuanxiang Zhang^2^, Changhua Wang^2^, Chung Hsien Wu^2^, Jiwen Jiang^2^, Fei Long^3^, Liyuan Han^4,^*, Qifeng Han^4^, Yi-Bing Cheng^1^, and Yong Peng^1,^*

^1^ State Key Laboratory of Advanced Technology for Materials Synthesis and Processing, Wuhan University of Technology, Wuhan 430070, P. R. China

^2^ Triumph Photovoltaic Materials Co., Ltd. No.1001 Yannan Avenue, High-Tech Development District, Bengbu, Anhui, P. R. China

^3^ College of Materials Science and Engineering, Guilin University of Technology, Guilin 541004, P. R. China

^4^ State Key Laboratory of Metal Matrix Composites, Shanghai Jiao Tong University, Shanghai 200240, P. R. China

*Corresponding authors. E-mail: [yongpeng@whut.edu.cn](mailto:yongpeng@whut.edu.cn) (Yong Peng); [han.liyuan@sjtu.edu.cn](mailto:han.liyuan@sjtu.edu.cn) (Liyuan Han)

**Supplementary Figures and Tables**

**
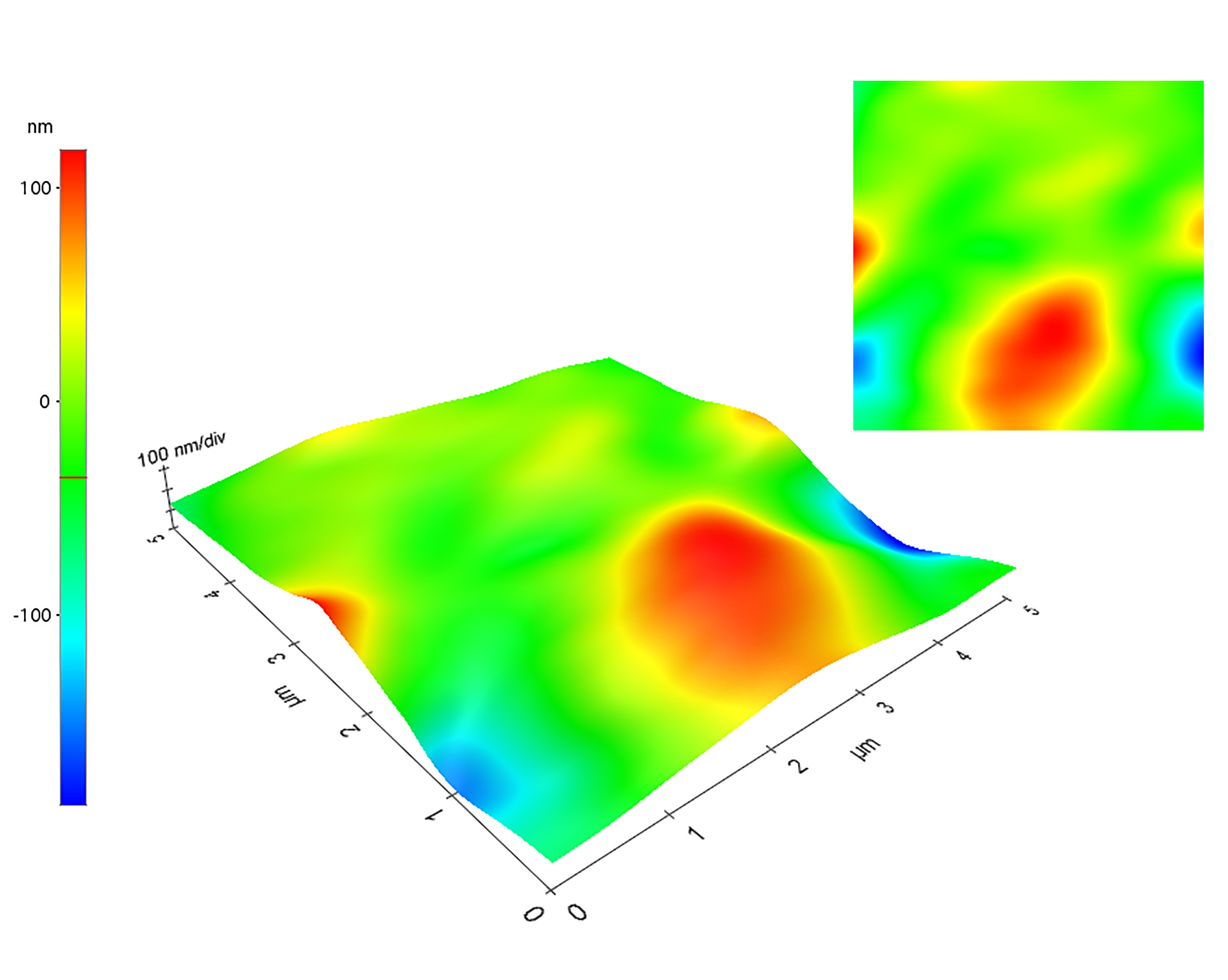
**

**Fig. S1** 5 µm × 5 µm AFM topography of surface PVK thin-film as-grown on CIGS


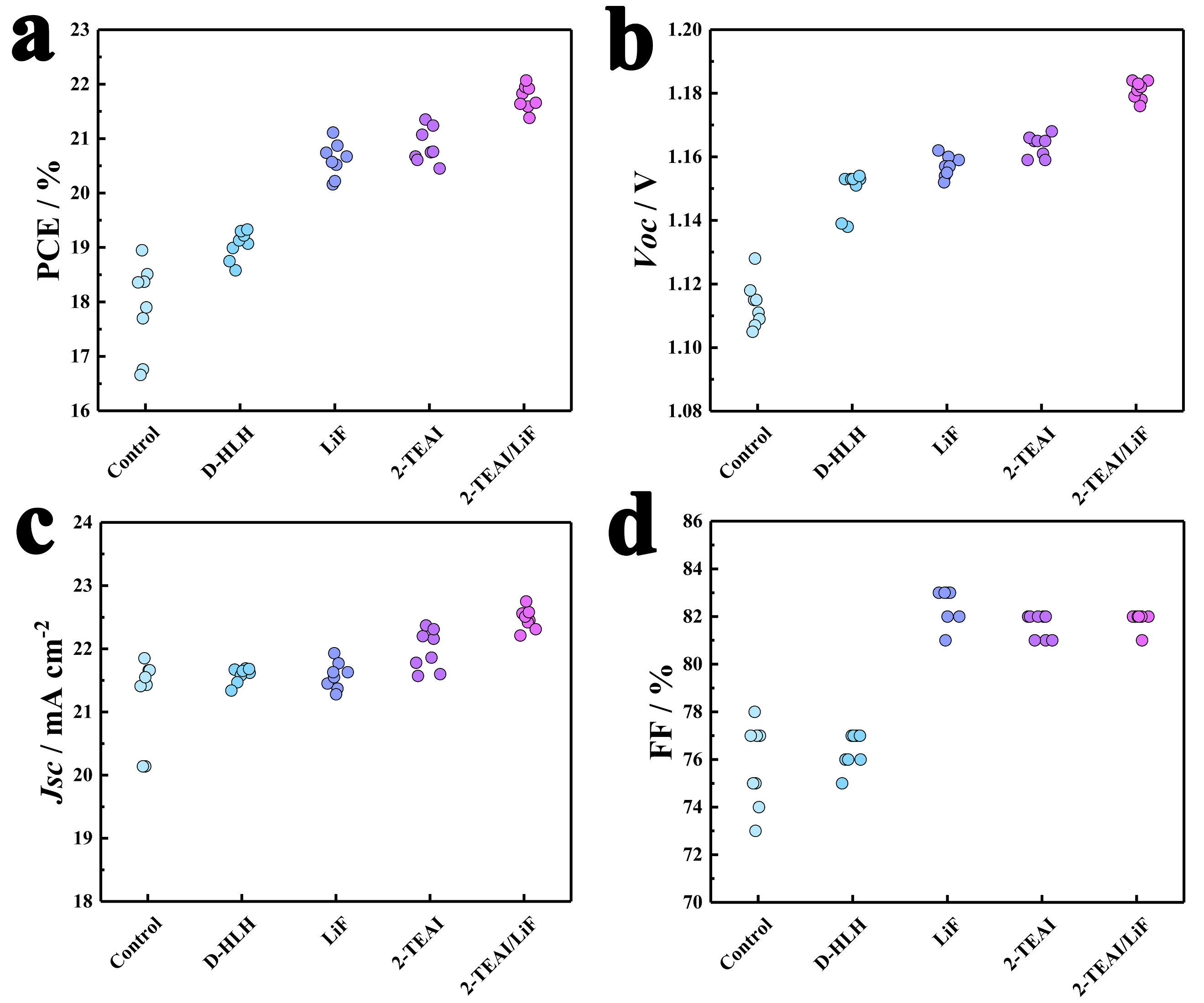


**Fig. S2** **a**-**d** Photo-voltaic (PV) parameter statistics of the control, D-HLH-treated, LiF-treated, and 2-TEAI-treated, and 2-TEAI/LiF-treated PSCs


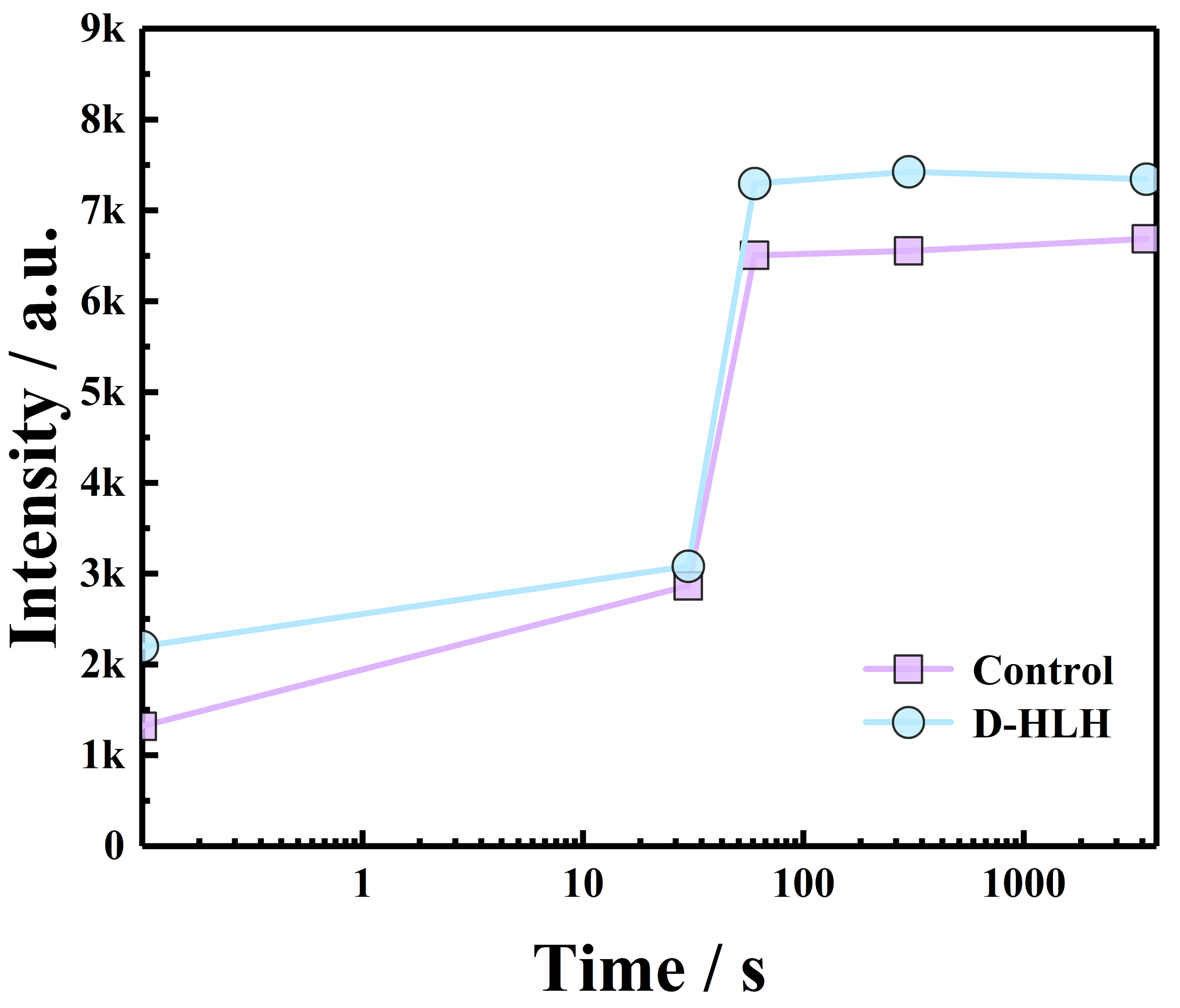


**Fig. S3** XRD intensity evolution for (100) crystal plane at 2θ = 14.00° during the annealing process


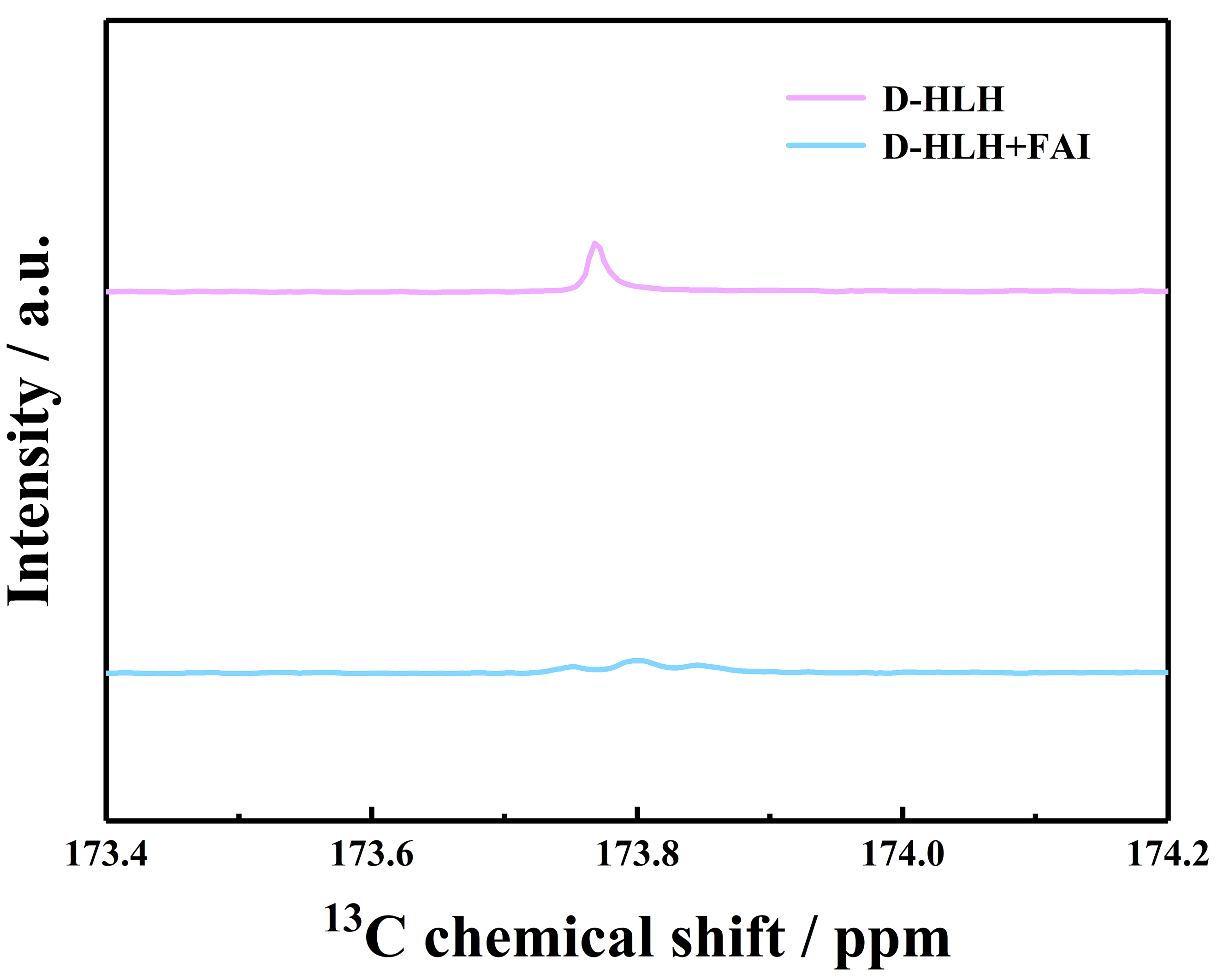


**Fig. S4** ^13^C NMR spectra of D-HLH and D-HLH/FAI


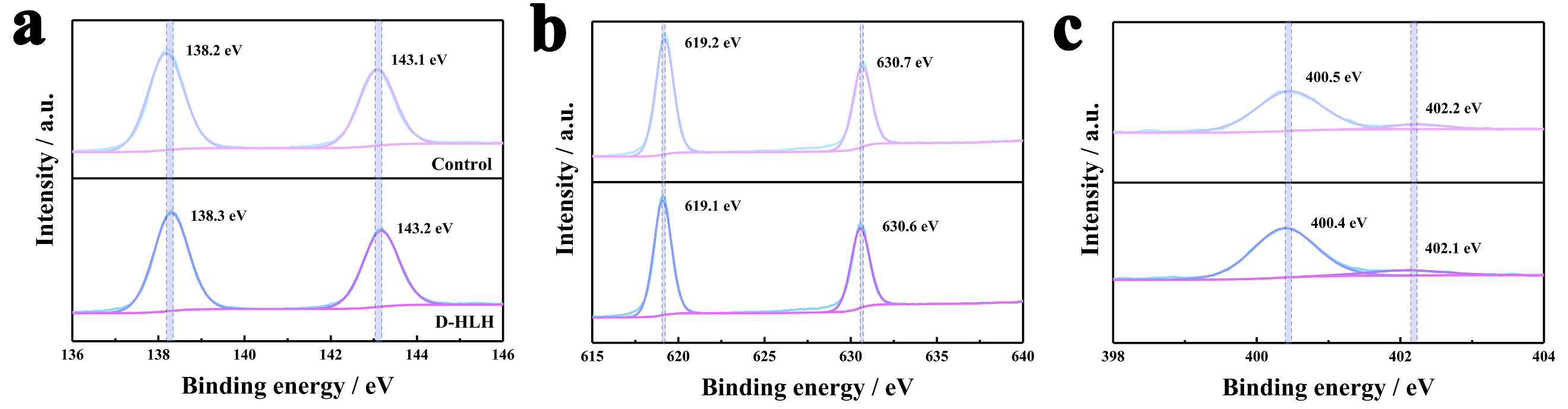
**Fig. S5** XPS spectra of **a** Pb 4f, **b** I 3d, and **c** N 1s of the control and D-HLH-tread films


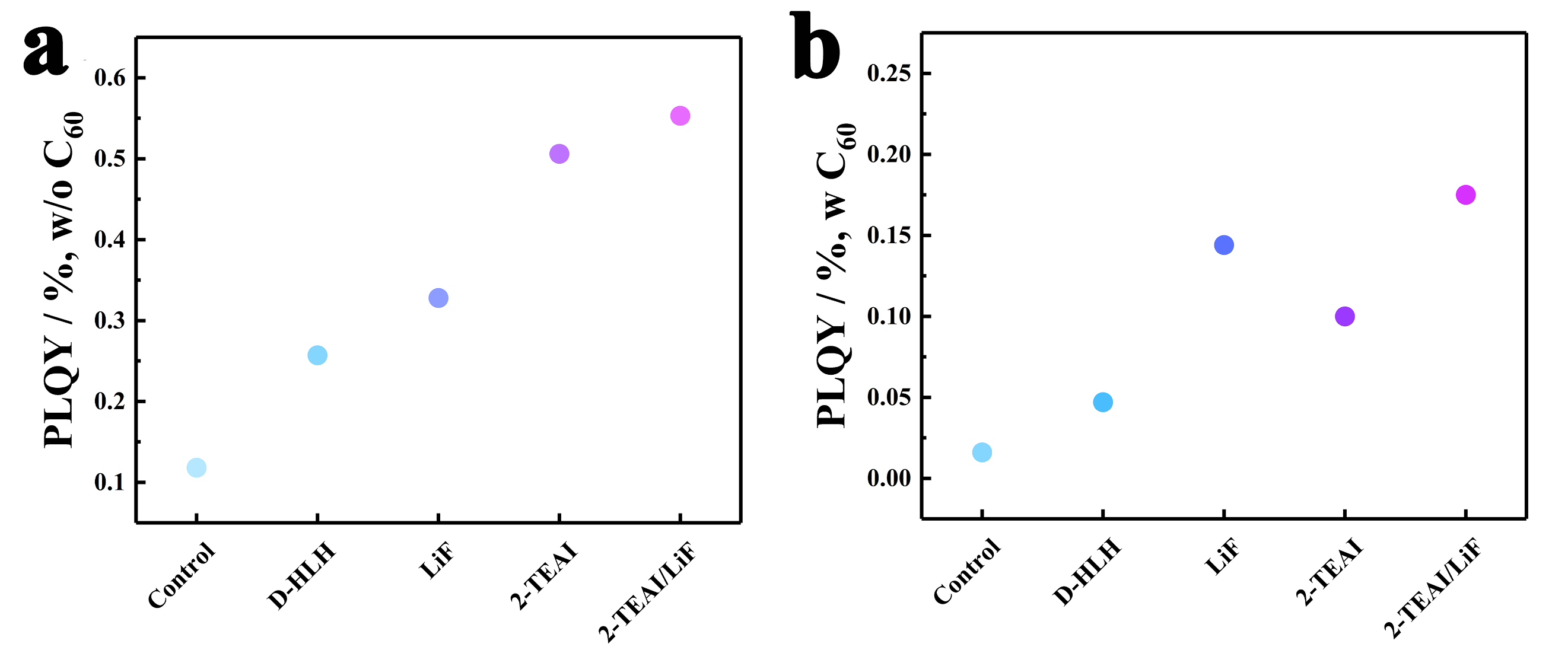


**Fig. S6** PLQY of the perovskite films on **a** ITO/SAM/PVK stack, and **b** ITO/SAM/PVK/passivation layer/C_60_ stack

The PLQY, which is directly related to the QFLS under the presumption that all PL emission results from the radiative recombination of free charges in perovskite film, can be represented by the following equation.

$$QFLS=K_{B}TIn(PLQY\times{J_{G}}/{J_{0,rad})}$$

where KB, and T represent Boltzmann constant and temperature, respectively. JG is the current density under illumination, and J0,rad is the dark radiative recombination saturation current density.

The following equations can be used to calculate the in accordance with the detailed balance theory:

$$J_{0,rad}=q\int_{0}^{\infty} {EQE}_{PV}\left( E \right)\emptyset_{BB}\left( E \right)dE$$

$$\emptyset_{BB}\left( E \right)=\frac{2\pi E^{2}}{h^{3}c^{2}}\frac{1}{\exp\left( \frac{E}{K_{B}T} \right)-1}$$

Where q, EQE_PV_, $\emptyset_{BB}$ , E, h, c represents elementary charge, photovoltaic external quantum efficiency, black-body radiative spectrum, photon energy, Planck constant, and light speed in vacuum, respectively.


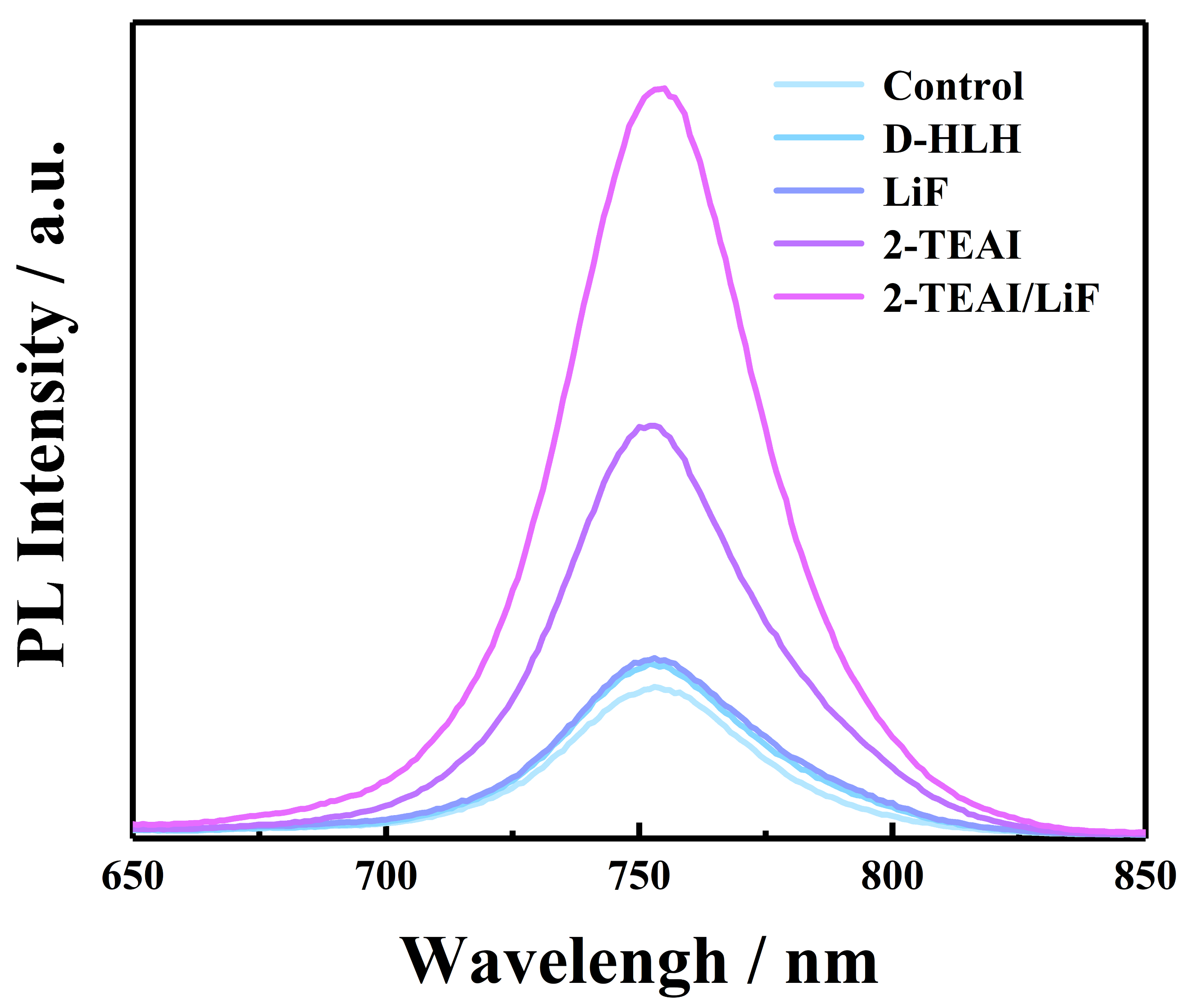


**Fig. S7** Steady-state PL spectra of the PVK films





**Fig. S8** Transient PL spectra of the glass/PVK/passivation layer /C_60_ stacks


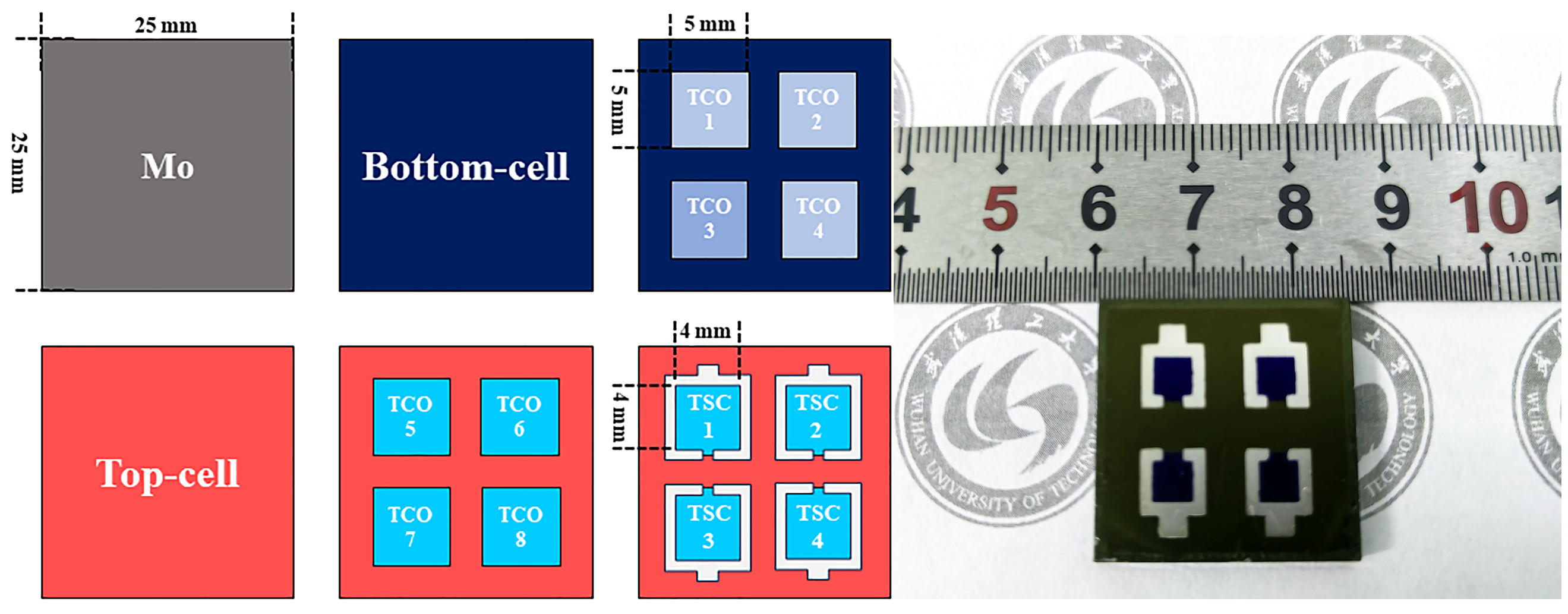


**Fig. S9** Schematic of the device layout used in the fabrication of the 2T PVK/CIGS TSCs. The final aperture area of 0.16 cm^2^ is defined by a black shadow mask used during *J-V* measurements


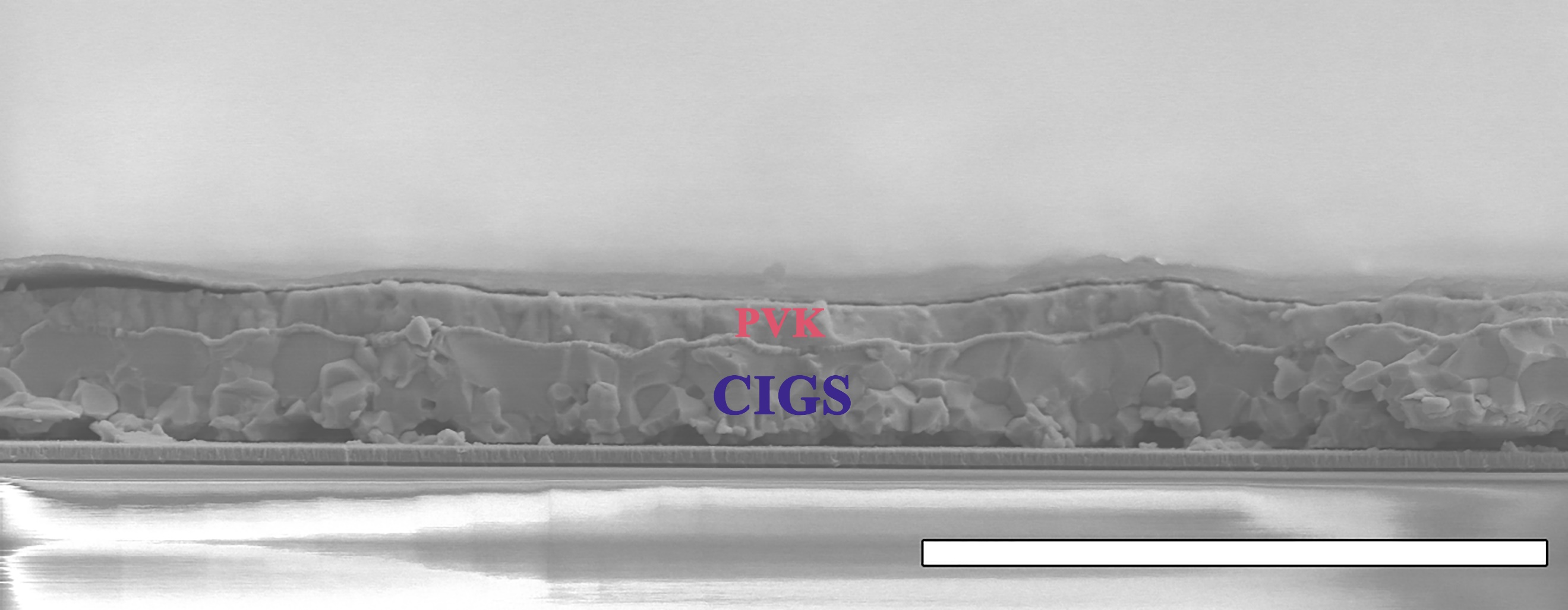


**Fig. S10** Cross-section SEM images the PVK/CIGS tandem solar cells. (Scale bar: 10 μm)


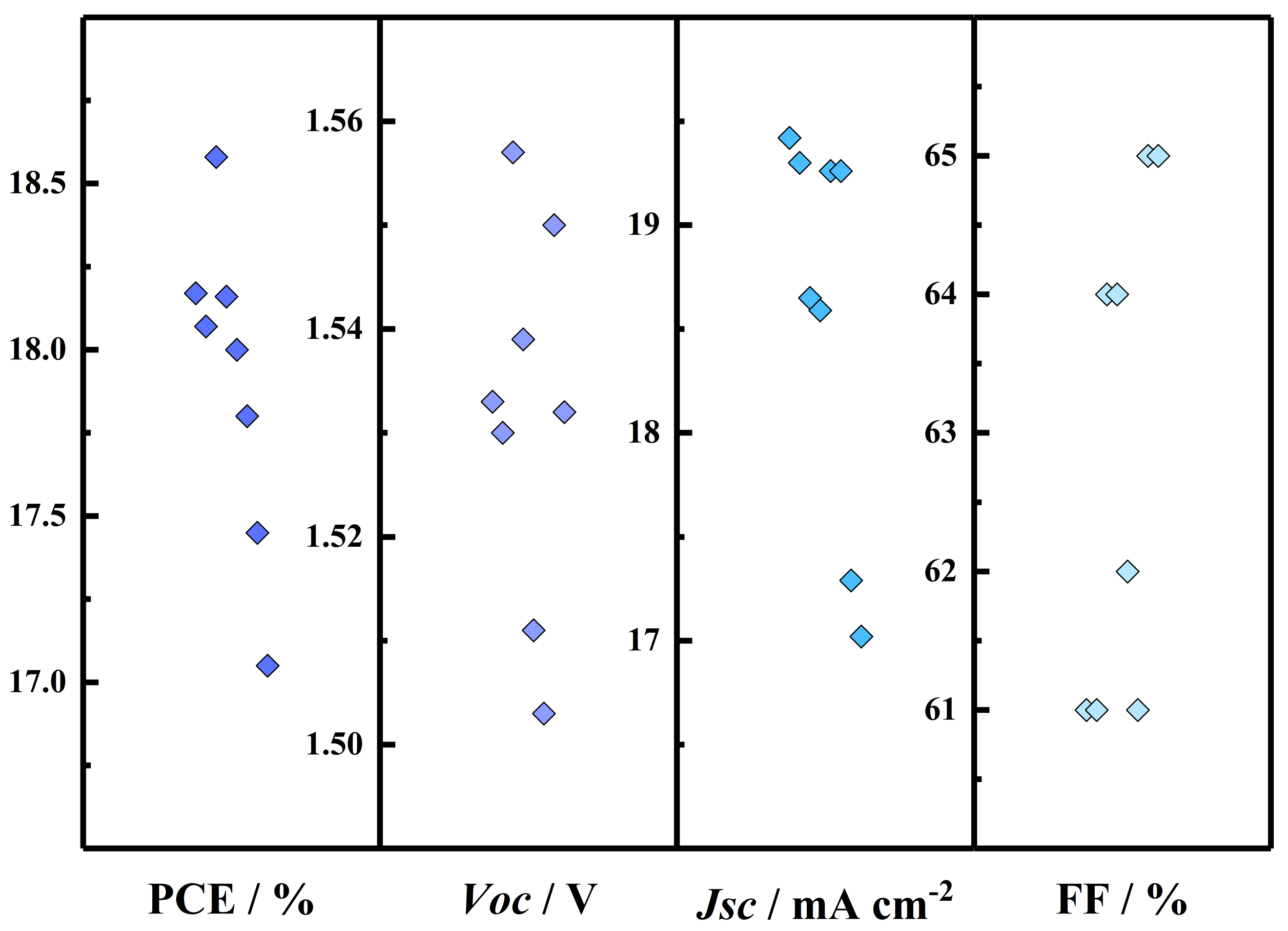


**Fig. S11** PV parameter statistics of PVK/CIGS tandem solar cells without NiO_x_


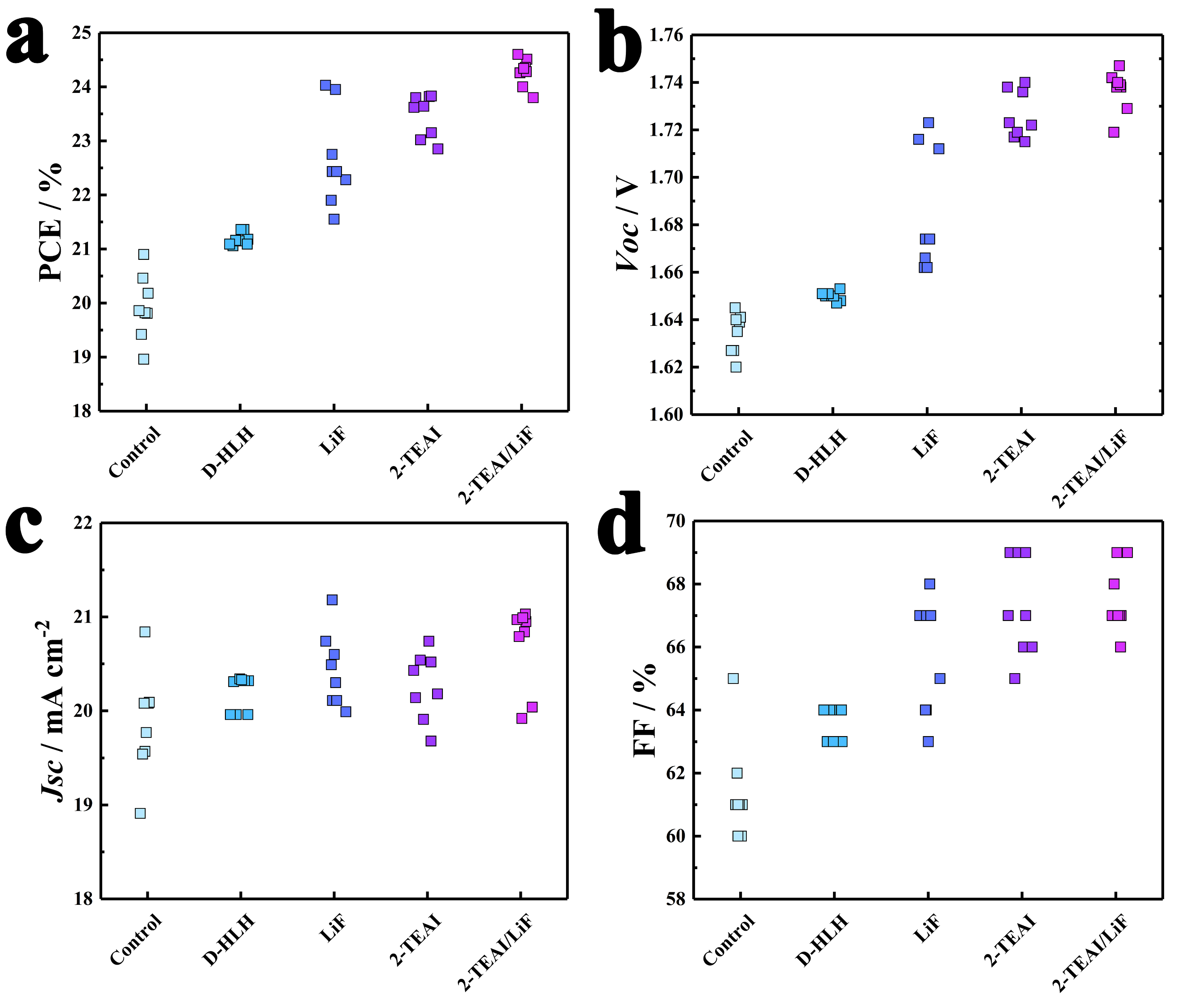


**Fig. S12** **a**-**d** PV parameter statistics of the control, D-HLH-treated, LiF-treated, and 2-TEAI-treated, and 2-TEAI/LiF-treated PVK/CIGS tandem solar. cells


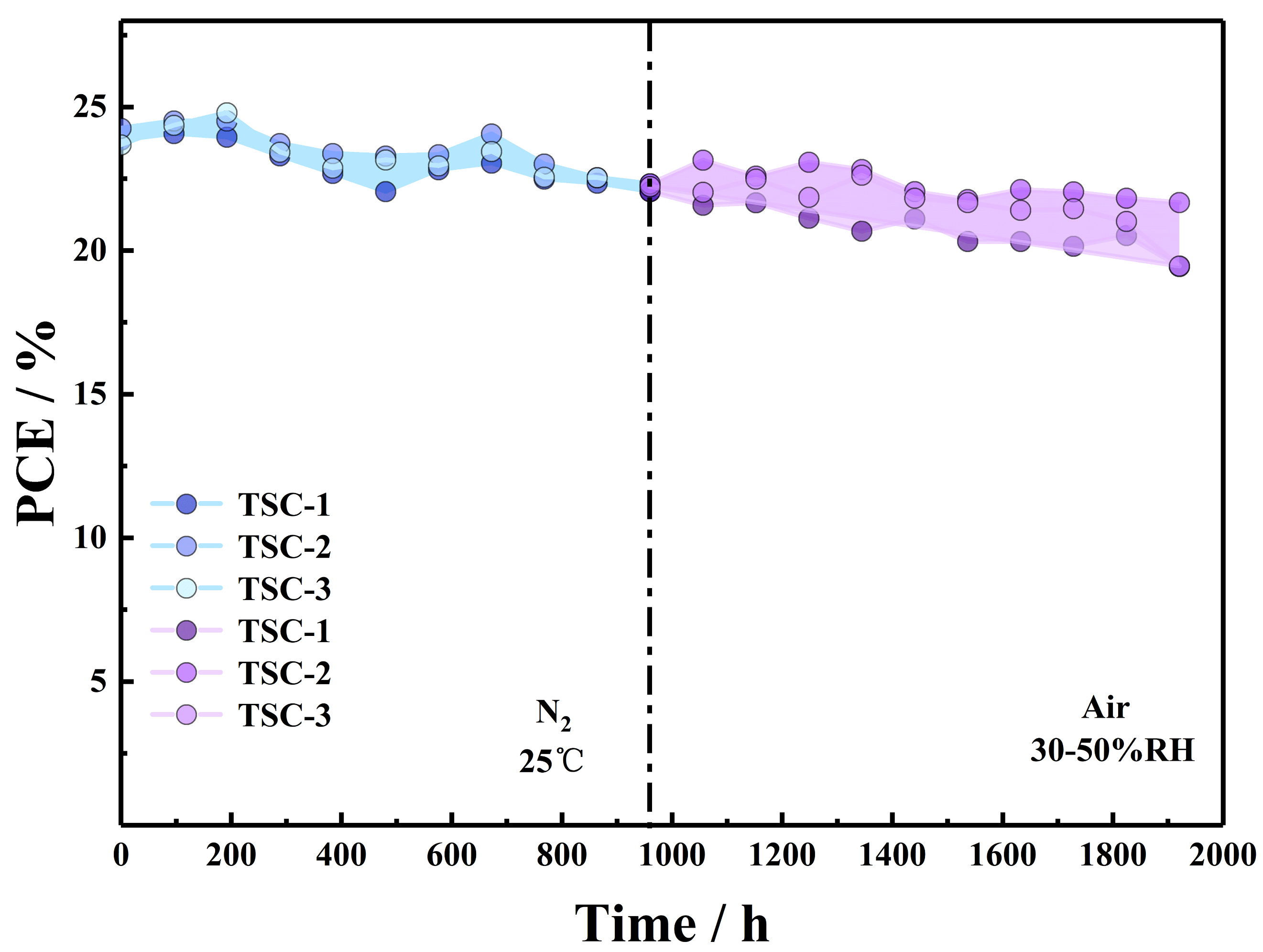


**Fig. S13** The stability of three unencapsulated tandem devices in nitrogen and air (30-50% RH) environment

**Table S1** Summary of 2-T PVK/CIGS tandem solar cells

| Year | Perovskite | Voc  (V) | Jsc  (mA / cm^2^) | FF  (%) | PCE  (%) | area  (cm^2^) | Refs. |
| --- | --- | --- | --- | --- | --- | --- | --- |
| 2015 | MAPbIBr | 1.45 | 12.7 | 56.6 | 10.9 | 0.4 | [S1] |
| 2017 | MAPbI_3_ | 1.346 | 12.9 | 63.5 | 11.03 | 0.5 | [S2] |
| 2018 | Cs_0.09_FA_0.77_MA_0.14_Pb(I_0.86_Br_0.14_)_3_ | 1.774 | 17.3 | 73.1 | 22.43 | 0.52 | [S3] |
| 2019 | Cs_0.05_(MA_0.17_FA_0.83_)_0.95_Pb(I_0.83_Br_0.17_)_3_ | 1.58 | 18 | 76 | 21.6 | 0.78 | [S4] |
| 2019 | Cs_0.05_(MA_0.17_FA_0.83_)_0.95_Pb(I_0.83_Br_0.17_)_3_ | 1.68 | 19.17 | 71.9 | 23.26 | 1.03 | [S5] |
| 2022 | Cs_0.05_(MA_0.23_FA_0.77_)Pb_1.1_(I_0.77_Br_0.23_)_3_ | 1.77 | 18.8 | 71.2 | 24.2 | 1.04 | [S6] |
| 2022 | Cs_0.05_MA_0.1_FA_0.85_Pb(I_0.9_Br_0.1_)_3_ | 1.59 | 19.4 | 75.5 | 23.5 | 0.5 | [S7] |
| 2024 | Cs_0.05_(MA_0.17_FA_0.83_)_0.95_Pb(I_0.83_Br_0.17_)_3_ | 1.742 | 20.97 | 67.3 | 24.6 | 0.16 | **This Wok** |

**Supplementary References**

1. T. Todorov, T. Gershon, O. Gunawan, Y. S. Lee, C. Sturdevant et al., Monolithic perovskite-cigs tandem solar cells via in situ band gap engineering. Adv. Energy Mater. **5**(23), 1500799 (2015). https://doi.org/10.1002/aenm.201500799
2. Y. H. Jang, J. M. Lee, J. W. Seo, I. Kim, D.-K. Lee, Monolithic tandem solar cells comprising electrodeposited CuInSe_2_ and perovskite solar cells with a nanoparticulate zno buffer layer. J. Mater. Chem. A **5**(36), 19439-19446 (2017). [https://doi.org/10.1039/C7TA06163C](https://doi.org/https://doi.org/10.1039/C7TA06163C)
3. Q. Han, Y.-T. Hsieh, L. Meng, J.-L. Wu, P. Sun et al., High-performance perovskite/Cu(In,Ga)Se_2_ monolithic tandem solar cells. Science **361**(6405), 904-908 (2018). [https://doi.org/10.1126/science.aat5055](https://doi.org/https://doi.org/10.1126/science.aat5055)
4. M. Jošt, T. Bertram, D. Koushik, J. A. Marquez, M. A. Verheijen et al., 21.6%-efficient monolithic perovskite/Cu(In,Ga)Se_2_ tandem solar cells with thin conformal hole transport layers for integration on rough bottom cell surfaces. ACS Energy Lett. **4**(2), 583-590 (2019). [https://doi.org/10.1021/acsenergylett.9b00135](https://doi.org/https://doi.org/10.1021/acsenergylett.9b00135)
5. A. Al-Ashouri, A. Magomedov, M. Roß, M. Jošt, M. Talaikis et al., Conformal monolayer contacts with lossless interfaces for perovskite single junction and monolithic tandem solar cells. Energy Environ. Sci. **12**(11), 3356-3369 (2019). [https://doi.org/10.1039/C9EE02268F](https://doi.org/https://doi.org/10.1039/C9EE02268F)
6. M. Jošt, E. Köhnen, A. Al-Ashouri, T. Bertram, Š. Tomšičet al., Perovskite/cigs tandem solar cells: From certified 24.2% toward 30% and beyond. ACS Energy Lett. **7**(4), 1298-1307 (2022). [https://doi.org/10.1021/acsenergylett.2c00274](https://doi.org/https://doi.org/10.1021/acsenergylett.2c00274)
7. M. A. Ruiz-Preciado, F. Gota, P. Fassl, I. M. Hossain, R. Singh et al., Monolithic two-terminal perovskite/cis tandem solar cells with efficiency approaching 25%. ACS Energy Lett. **7**(7), 2273-2281 (2022). <https://doi.org/> <https://doi.org/10.1021/acsenergylett.2c00707>
